# Full endoscopic anterior intrapelvic plate osteosynthesis: a cadaveric feasibility study

**DOI:** 10.1007/s00402-022-04346-z

**Published:** 2022-01-18

**Authors:** Maximilian J. Hartel, Gerrit Althoff, Stefan Wolter, Benjamin Ondruschka, Eric Dietz, Karl-Heinz Frosch, Darius M. Thiesen

**Affiliations:** 1 Department of Trauma and Orthopaedic Surgery, University Medical Center Hamburg-Eppendorf, Martinistraße 52, 20246 Hamburg, Germany; 2 Department of Trauma Surgery, Orthopaedics and Sports Traumatology, BG Hospital Hamburg, Hamburg, Germany; 3 Department of General, Visceral and Thoracic Surgery, University Medical Center Hamburg-Eppendorf, Hamburg, Germany; 4 Institute of Legal Medicine, University Medical Center Hamburg-Eppendorf, Hamburg, Germany

**Keywords:** Endoscopic surgery, Pelvic ring fracture, Acetabular fracture, Plate osteosynthesis, Minimally invasive surgery, Cadaver study

## Abstract

In this investigation, it was assumed that it must be possible to visualize the intrapelvic aspect as accustomed by pelvic surgeons using the anterior intrapelvic (modified Stoppa) approach. Moreover, it was hypothesized, that plate mountings will not only be possible for the symphysis but also at the supra- and infrapectineal aspect as well as to the posterior column. Ten anonymized cadaveric specimens were included in this study. A standard laparoscopic totally extraperitoneal (TEP) approach was used. A total of 10 landmarks were defined that are usually within reach in the open anterior intrapelvic (AIP) approach. Moreover, five different plate mountings were tested. The locations were chosen in accordance with the indication spectrum suitable for open surgery through the traditional AIP approach. It was possible to gain intrapelvic visibility in seven of ten cases. In all of those seven cases, it was technically possible to place plates to the symphysis, superior pubic ramus, as well as longer anterior column plates up to the aspect posterior of the acetabulum. In the last four of the seven cases, it was possible to mount plates to the infrapectineal aspect as well as the posterior column, too. The team, previously trained in arthroscopic surgical techniques as well as pelvic trauma surgery, observed a steep learning curve. This investigation demonstrated, that endoscopic anterior intrapelvic plate osteosynthesis was feasible in the majority of the cases in a series of ten cadaveric models. New instruments will be needed such as extra-long rasp elevators, ball-spikes as well as devices to hold and position plates and extra-long self-holding screwdrivers. With these, endoscopic pelvic surgery will likely be a realistic option for selected pelvic trauma cases in the future.

## Introduction

In the 1960s Professor Emile Letournel developed the two-column concept as well as his classification of acetabular fractures [1]. The approaches he suggested helped surgeons to accomplish reproducible results [2, 3]. Unlike the theoretical groundwork which has withstood the decades and is still a key pillar of surgical decision making to date, especially the extended approaches were decreasingly used by pelvic surgeons over time [4]. In the early nineties, Cole and Bolhofner as well as Hirvensalo et al. described a less invasive anterior approach for pelvic and acetabular fracture cases [5, 6]. This anterior intrapelvic approach was also called the “Stoppa approach” as it resembles the technique described by Stoppa et al. for the use of hernia repair [7, 8].

This approach is also established at the investigating site, due to its wide range of applications and its extensibility to the traditional ilioinguinal approach. Its comparably low soft tissue damage makes it a suitable option in elderly patients, too [9, 10]. Recent research as well as case reports showed, that a further minimization using endoscopic techniques may evolve to be another option in the future [11–15]. Rubel et al. first described a technique of plate and screw osteosyntheses to the anterior pelvic ring in a series of two in 2002 [16]. In 2019, Küper and colleagues described an endoscopic technique for plate osteosyntheses to the symphysis and anterior pelvic ring [12]. Next, Trulson et al. showcased in their cadaveric series of four, that it was feasible to visualize the nine landmarks around the acetabulum and demonstrated that a suprapectineal plate osteosynthesis of the acetabulum could be performed endoscopically [14]. Finally, a novel transperitoneal approach was described by Küper et al. providing superior space for intrapelvc preparation [13].

To further explore this topic, a total of ten consecutive cadaveric specimens was analysed. It was hypothesized, that it will be possible to reproducibly visualize the intrapelvic aspect as accustomed by pelvic surgeons using the anterior intrapelvic (AIP) approach in a majority of these cases. Moreover, it was hypothesized, that plate mountings will not only be possible for the symphysis and suprapectineal aspect but also at the infrapectineal aspect as well as to the posterior column.

## Methods

Ten anonymized cadaveric specimens were included in this study. As a prerequisite, a consent to body donation for scientific and educational purposes needed to be available. This study was reviewed by the local Ethics Committee (process number: WF-048/21). All procedures were carried out in accordance with the 1964 Helsinki Declaration and its later amendments and comparable ethical standards.

### Technique

**Fig. 1:**
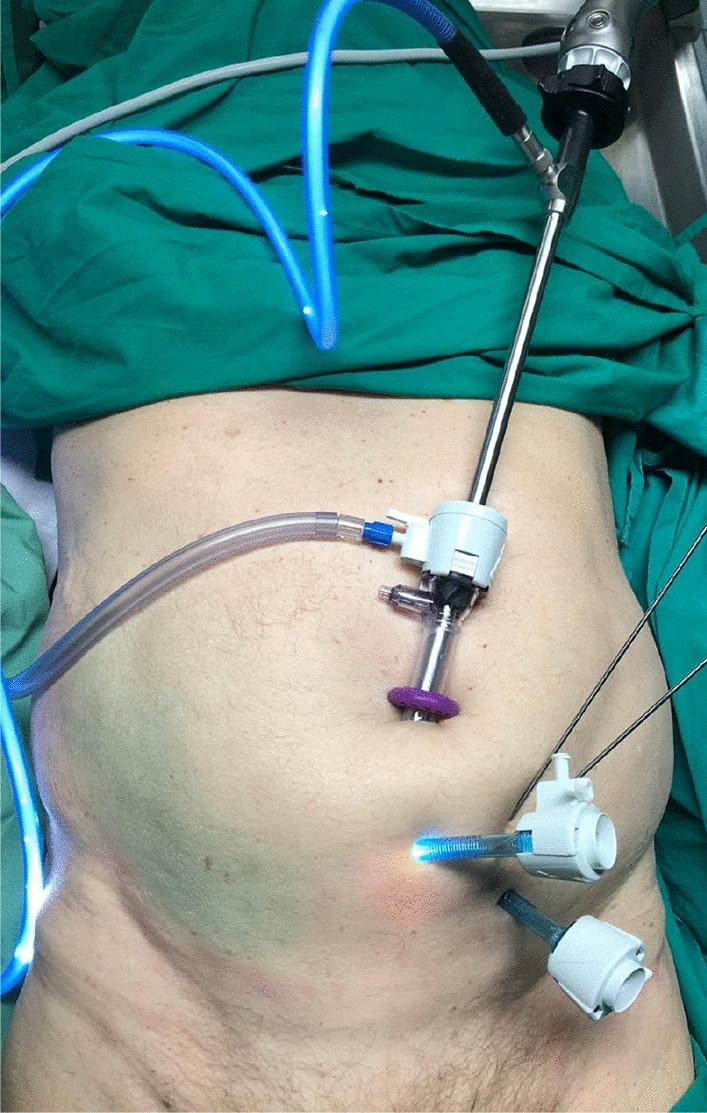
Shows a typical trocar setup used in this study

All operations were performed in the supine position. A standard laparoscopic totally extraperitoneal approach (TEP) was used in all cases starting with a median infraumbilical incision of 1.5–2 cm. The anterior rectus sheath was identified and incised with care without reaching the peritoneal level. Next, the rectus abdominis muscle was mobilized and lifted in superior-lateral direction using a narrow Langenbeck retractor followed by blunt dissection in the direction of the symphysis to create a cavity. A balloon trocar (PDB® balloon dissector, Medtronic; Dublin, Ireland) was introduced and gradually inflated under laparoscopic visual control. The balloon trocar was replaced by a standard 10 mm trocar. As usual in open surgery, starting at the symphysis, the retropubic space of Retzius is developed bluntly. Two more (trans- and pararectal) portals were established under direct visualization to avoid epigastric vascular damage (Fig. 1). As orthopedic trauma surgeons with training in arthroscopy, using a long 1.6 mm k-wire for portal planning and triangulation was found to be useful. After finalizing, the basic dissection landmarks were started to be identified. The rest of the dissection was carried out in a similar way to open surgery with the difference that a long narrow raspatorium and an endoscopic expandable retractor were used for dissection of the iliopsoas and obturator internus muscles as well as the obturator nerve instead of a regular cobb elevator [7]. In situations where even the long rasp elevator was found to be too short, a long screwdriver (Stryker Pelvic Pro® System, Stryker Corporation; Kalamazoo, USA) needed to be misused instead. After finishing the dissection, it was noted whether all necessary landmarks were reached. Afterwards, the plate placement was started. Short plates were introduced through a 10 mm trocar. Longer, curved plates (12 hole), needed to be directly introduced through one of the portals. Percutaneous long K-wires were used for preliminary fixation of the plates. To prevent soft tissue damage, the wires were inserted and removed in oscillating mode. The drilling and insertion of the screws was performed through the trocars under endoscopic vision to prevent soft tissue damage. A suture was used to secure the screws to the screwdriver similar to the technique used in minimally invasive plate osteosynthesis (MIPO). Long drill bits and screwdrivers needed to be used for the technique (Stryker Pelvic Pro®).

### Hypothesis part 1—anatomical landmarks

A total of ten landmarks were defined that are usually within reach in the open AIP approach. In cases where all landmarks have reached a visualization similar to the full AIP approach was assumed. The landmarks were

1. Symphysis
2. Superior pubic ramus
3. Corona mortis
4. Obturator nerve
5. Obturator foramen
6. External iliac vessels
7. Iliosacral joint
8. Greater sciatic foramen
9. Ischial spine (palpable)
10. Lesser sciatic foramen (palpable)

### Hypothesis part 2—feasibility of different plate mountings

**Fig. 2:**
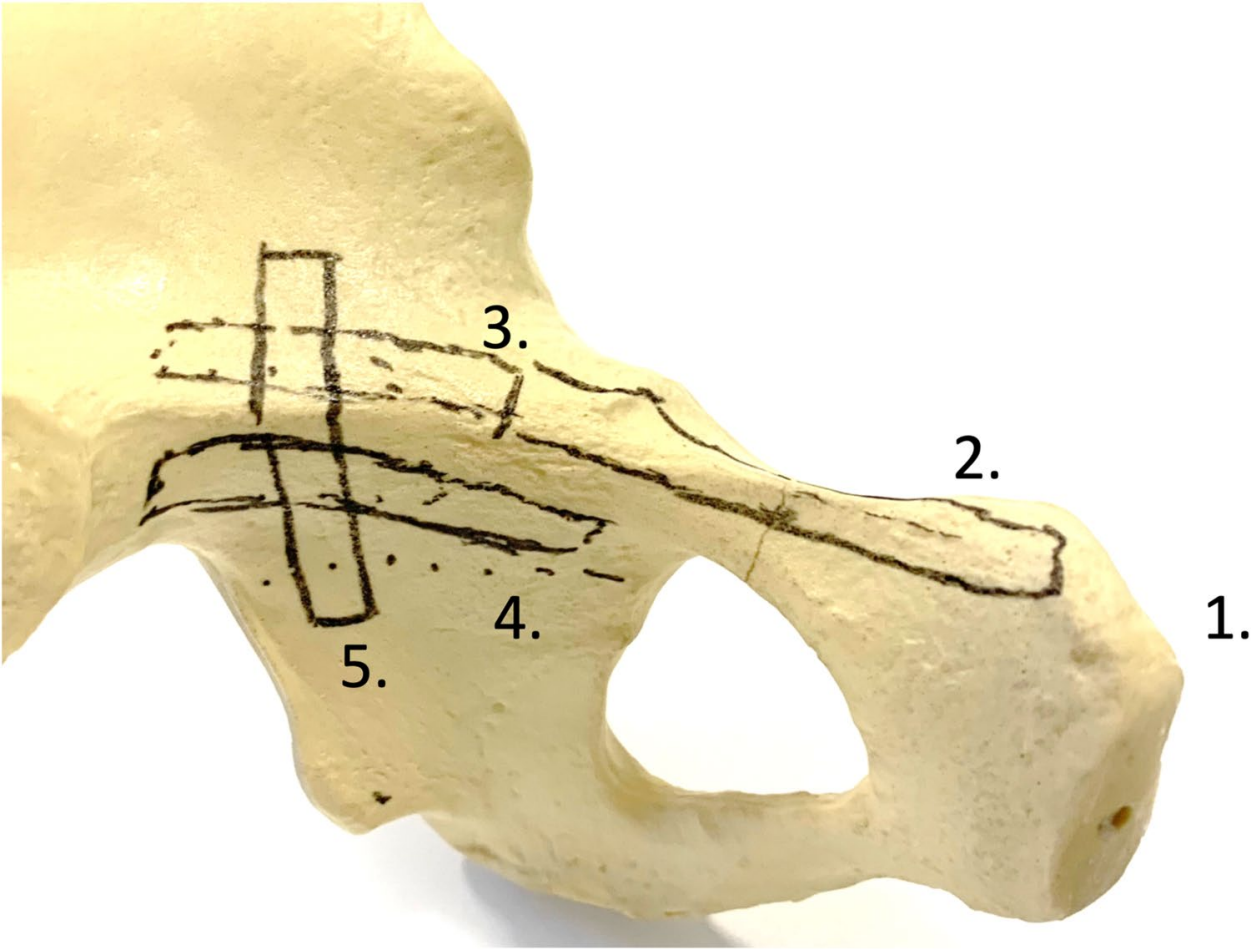
Shows the hypothetic localizations of possible plates laparascopicallly delivered. The intended plate positions were drawn on the pelvic bone model. Plate locations: (1) Symphysis (6-hole plate). (2) Superior pubic ramus (6-hole plate). (3) Suprapectineal to pelvic brim plate (12-hole plate). (4) Infrapectineal plate (6 hole plate). (5) Plate to the posterior column (6-hole plate)

It was tested whether five different plate mountings were possible. The locations were chosen in accordance with the indication spectrum suitable for open surgery through the AIP approach (Fig. 2) [5, 7]. Small fragment reconstruction plates and conventional 3.5 mm screws were utilized (Arthrex 3.5 mm reconstruction plates, Arthrex; Naples, USA). The plates were manually contured to fit the individual anatomy of the corpses.

Plate locations:

1. Symphysis (6-hole plate)
2. Superior pubic ramus (6-hole plate)
3. Suprapectineal to pelvic brim plate (12-hole plate)
4. Infrapectineal plate (6 hole plate)
5. Plate to the posterior column (6-hole plate)

## Results

### Sample description

Ten cadaveric specimens were included in this study. Four of them were female (40%). The median age amounted to 74 years (IQR 66–78.5, range 55–84). The median BMI was 26.1 kg/m^2^ (IQR 20.2–26.3, range 16.7–42.0). Two of the samples had previous abdominal surgery (one with median laparotomy and one with a history of hernia repair with mesh implanted): due to anonymization, it was impossible to obtain the exact history. In three cases (30%) the investigators decided to abort the procedures: one specimen presented with a BMI of 42.0 kg/m^2^. In this case, it was technically impossible to carry out the procedure. A second case exhibited soft tissues too disintegrated by post mortem processes for a meaningful procedure. In the third case with a median laparotomy scar it was hardly possible to obtain intrapelvic visibility let alone allowing for plate placement.

### Hypothesis part 1—anatomic landmarks

**Fig. 3:**
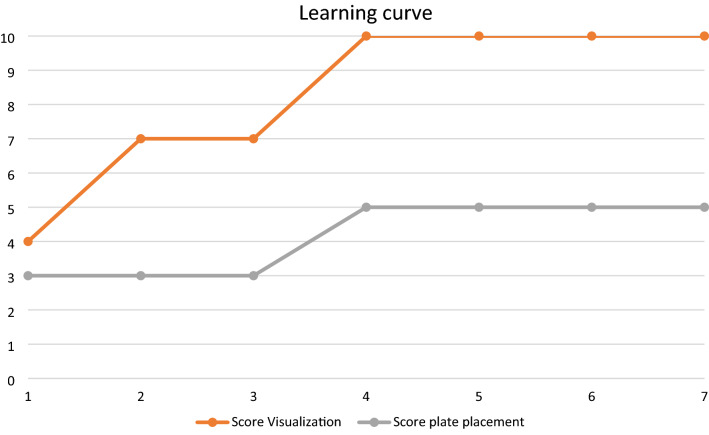
Shows the amount of anatomical landmarks reached (maximum 10) as well as plate configurations implanted (maximum 5), that could be achieved in the chronological consecutive order in the technically feasible subgroup of seven cases

**Fig. 4:**
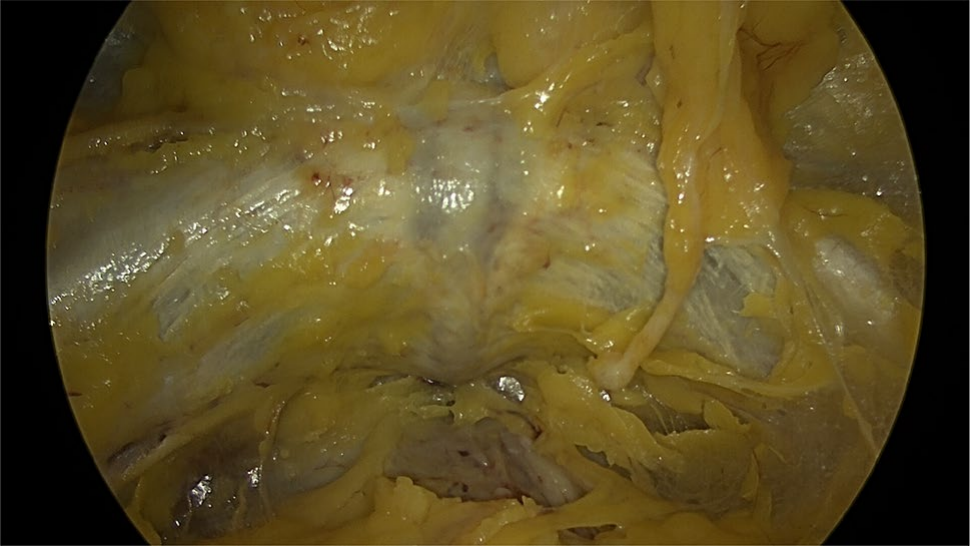
Shows an overview of the symphysis using the endoscopic approach

**Fig. 5:**
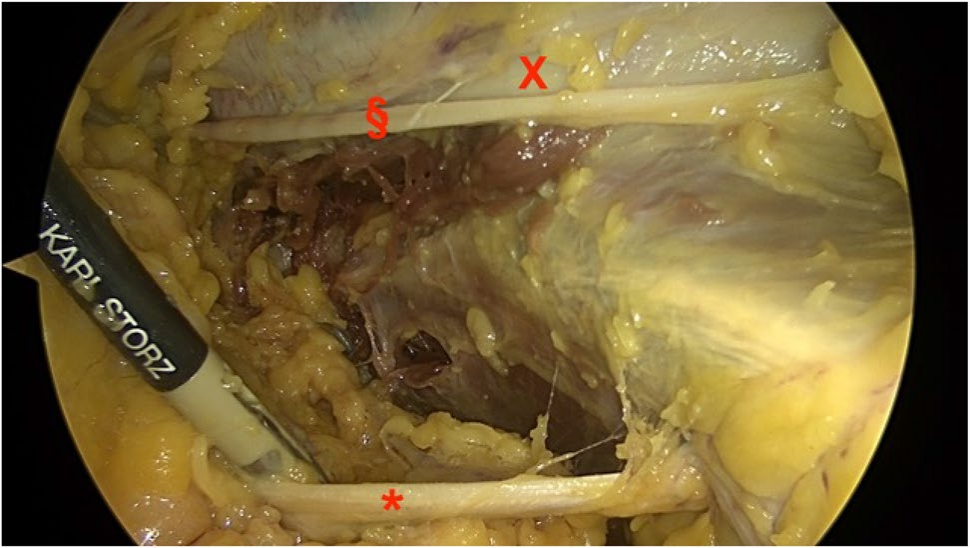
Shows the aspect of the pelvic brim with the obturator nerve inferior, running into the obturator foramen (*), the external iliac vessels superior (x) and the genitofemoral nerve (§) alongside the vessels

It was possible to gain sufficient intrapelvic visibility in seven of ten cases. The team, previously trained in arthroscopic surgical techniques, observed a steep learning curve. Figure 3 shows the amount of anatomical landmarks reached (maximum 10) as well as plate configurations implanted (maximum 5), that could be achieved in the chronological consecutive order in the technically feasible subgroup of seven cases. Figures 4 and 5 demonstrate typical views to the symphysis as well as along the pelvic brim.

### Hypothesis part 2—feasibility of different plate mountings

**Fig. 6:**
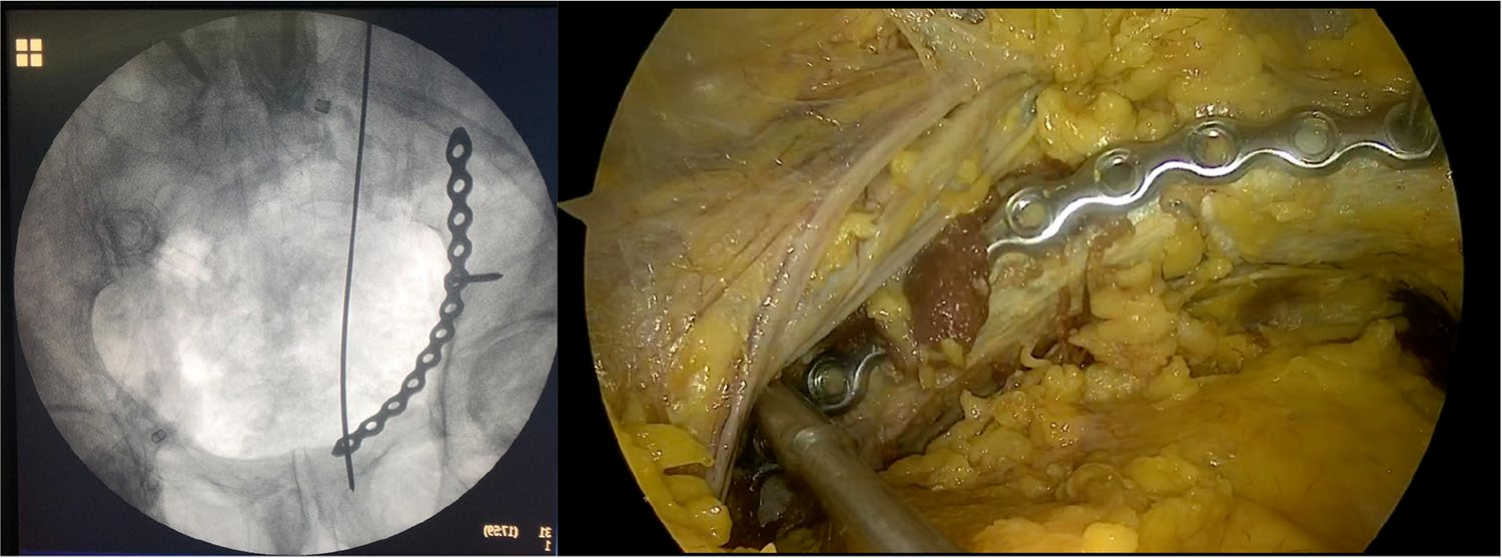
Shows an example of a plate with the corresponding radiograph mounted to the suprapectineal aspect extending next to the sacroiliac joint. In this case, even the most posterior hole could be visualized and reached despite an extended hip due to the body stiffness regularly encountered in the chemically unfixed cadavers investigated as remaining rigor mortis

**Fig. 7:**
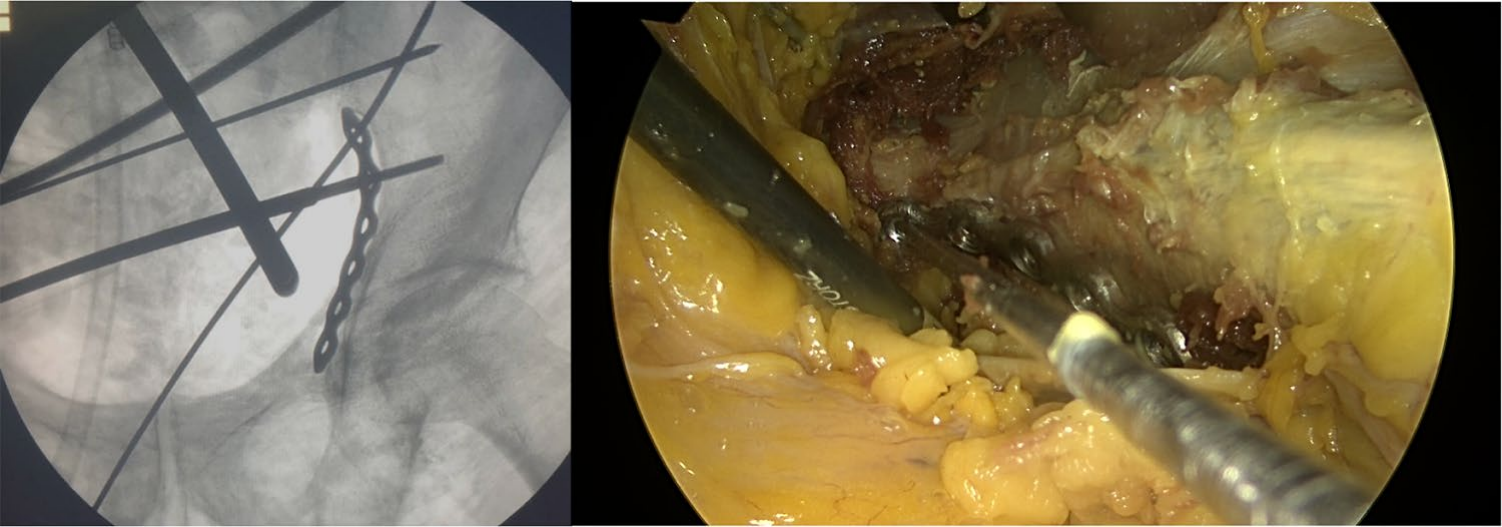
Shows an example of a plate placed to the aspect below the pelvic rim with a corresponding radiograph

**Fig. 8:**
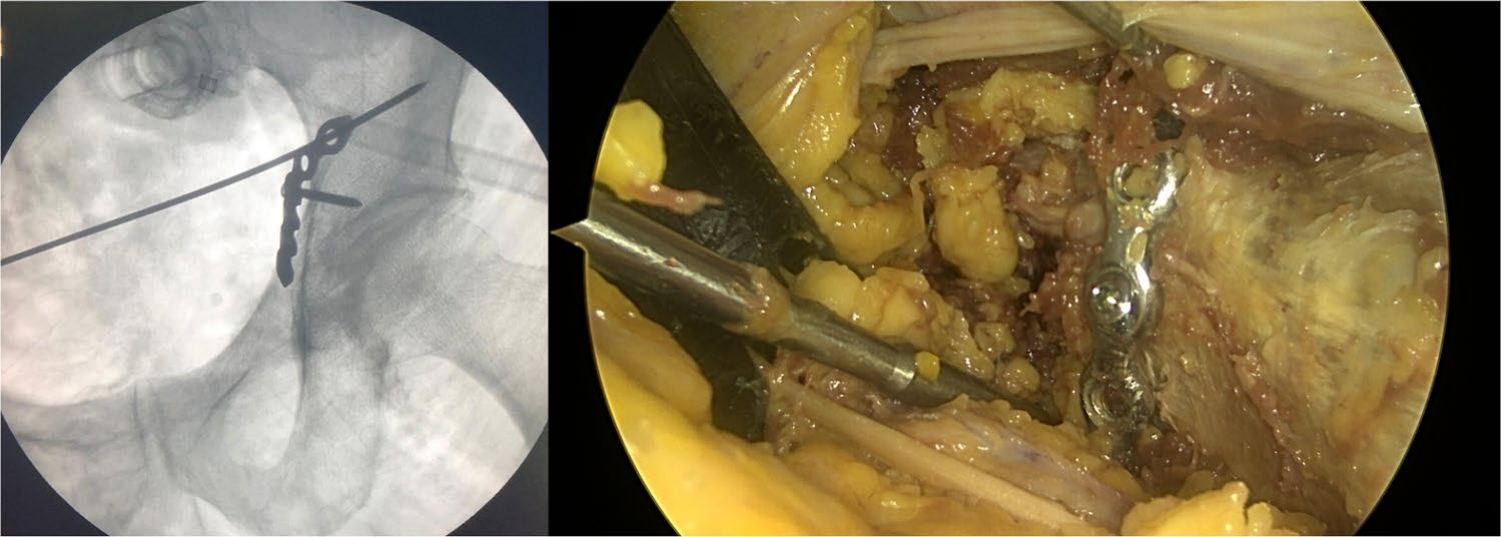
Shows an example of a plate positioned to the posterior column with a corresponding radiograph. The screwdriver is pointing into the greater sciatic notch indicating the posterior border of the posterior column

As mentioned above, it was possible to gain anterior intrapelvic visibility in seven of ten cases. In all of these, it was technically possible to place plates to the symphysis, superior pubic ramus, as well as longer suprapectineal plates to the upper pubic ramus extending the pelvic rim (linea terminalis) to the aspect posterior of the acetabulum. In the first three of these seven cases it was not feasible to place plates to the infrapectineal aspect (below pelvic brim) as well as the posterior column from inside but for the following four cases placement of plates had been done even in these anatomical regions (Fig. 3). Figures 6, 7 and 8 show examples of plates positioned to the pelvic brim, to the infrapectineal aspect as well as to the posterior column.

### Technical issues

Several technical issues were encountered in this investigation: very quickly, the fact needed to be accepted, that conventional instruments, even longer versions, used in pelvic surgery were not reasonably applicable most of the time. Extra-long rasp elevators, drill bits and self-holding screwdrivers are necessary for the future. The team learned during the study progress, that a transrectal 10 mm trocar is needed for better visualization of the infrapectineal and especially, the posterior column aspect. The infraumbilical portal is used for the endoscopic expandable retractor. Moreover, handling the plates within the pelvis was demanding at times. Next, longer, curved plates do not fit through standard 10 mm trocars. It was necessary to quickly remove one of the trocars, insert the plate and reinsert the trocar without losing all the intrapelvic gas. Therefore, the contouring process of the plates was more time consuming, when compared with an open procedure.

## Discussion

This feasibility study demonstrated, that endoscopic anterior intrapelvic plate osteosynthesis is feasible in the majority of the cases in a series of ten cadaveric models.

A limited amount of literature, mostly case reports and small case series exists on the topic of endoscopic pelvic surgery to date [16–19]. Trulson and colleagues first reported a feasibility study in four cadaveric specimens for intrapelvic visualization and plate placement to the superior acetabular aspect [14]. The same research group published a technique on endoscopic plating of the symphysis as well as first clinical results [12]. In a technical note, Küper reported a laparoscopic preperitoneal approach which is similarly used in laparoscopic pelvic lymphadenectomy. This technique seems to provide superior space for instrumentation, when compared to a total extraperitoneal approach [13]. However, the strictly preperitoneal AIP (or modified Stoppa) approach has replaced the ilioinguinal approach as a standard anterior approach in recent years [20]. Orthopedic trauma surgeons trained in using the AIP approach may therefore encounter difficulties to adapt to transperitoneal techniques. The concept of this study was to analyze whether the anatomical exposure and technical capabilities usually achieved by the open AIP approach could also be met with a full-endoscopic technique. Zeng and colleagues published a case report of an unstable, moderately displaced anterior column acetabular fracture. A percutaneous procedure was not possible. Therefore, a laparoscopic approach was chosen [11]. They additionally used 3D printing technology to pre-contour a reconstruction plate and predetermine screw lengths. They reported an uncomplicated surgery and further course. In open acetabular surgery, 3D printing technology has demonstrated its usefulness, before [21]. Next to laparoscopic techniques at the pelvis, 3D printing technology may likely be one of the pillars of technical advances in future pelvic and acetabular surgery [22].

The current investigation has several limitations: first, a lower case number needed to be elected for ethical reasons. Next, the cadaveric study setting itself needs mentioning: first, the specimens used had intact osseous and soft tissues (apart from the ones with previous surgical scars). Intrapelvic fracture hematoma will likely make endoscopic surgery more demanding. Moreover, the feasibility of endoscopic fracture reduction without the option of introducing reduction clamps will need separate scientific attention in the future. It can be assumed, that endoscopic procedures will not be recommended for highly dislocated pelvic and acetabular fractures at first. Due to stiff joints and soft tissues as rigor mortis the investigators were unable to perform the intervention with the hip flexed as usual in anterior intrapelvic surgery. In a living patient, hip flexion may further optimize intrapelvic visibility due to relaxation of the vessels, nerves as well as the psoas muscle and tendon.

It can be concluded, that endoscopic anterior intrapelvic surgery and plate osteosynthesis was feasible in the non-obese, non-operated and non-putrefacted part of a cadaveric specimen series of ten. For reliable results in actual patients, new instruments will be needed such as extra-long rasp elevators, ball-spikes as well as devices to hold and position plates and extra-long self-holding screwdrivers.
